# Reducing False Alarms of Intensive Care Online-Monitoring Systems: An Evaluation of Two Signal Extraction Algorithms

**DOI:** 10.1155/2011/143480

**Published:** 2011-02-27

**Authors:** M. Borowski, S. Siebig, C. Wrede, M. Imhoff

**Affiliations:** ^1^Fakultät Statistik, Technische Universität Dortmund, 44227 Dortmund, Germany; ^2^Universitätsklinikum Regensburg, 93042 Regensburg, Germany; ^3^Helios Klinikum Berlin-Buch, 13125 Berlin, Germany; ^4^Abteilung für Medizinische Informatik, Biometrie und Epidemiologie, Ruhr-Universität, Bochum, 44801 Bochum, Germany

## Abstract

Online-monitoring systems in intensive care are affected by a high rate of false threshold alarms. These are caused by irrelevant noise and outliers in the measured time series data. The high false alarm rates can be lowered by separating relevant signals from noise and outliers online, in such a way that signal estimations, instead of raw measurements, are compared to the alarm limits. This paper presents a clinical validation study for two recently developed online signal filters. The filters are based on robust repeated median regression in moving windows of varying width. Validation is done offline using a large annotated reference database. The performance criteria are sensitivity and the proportion of false alarms suppressed by the signal filters.

## 1. Introduction

In intensive care, the condition of a patient is supervised by online-monitoring systems which measure several vital signs with a high sampling rate of up to one observation per second. These devices produce different types of alarms to alert the clinical staff. Most frequently these alarms are so-called simple threshold alarms, which are given when the measured values of a vital sign lie outside of specified alarm limits. The high rate of irrelevant threshold alarms, caused by artefacts and short fluctuations, is a well-known problem and shown in several studies; see [1] or [2], for instance. High false alarm rates may lead to a dangerous desensitization of medical staff toward alarms and, thus, dramatically reduce the effective sensitivity of the entire alarm system [3–5]. Moreover, alarm limits may be set inadequately wide or alarms disabled completely. Obviously, online-monitoring systems are in need of improvement with respect to the high rates of false positive alarms.

Several approaches for improved alarm systems have been proposed. For instance, median filters can be used to eliminate noise and artefacts [6]; in [7], a method based on control charts is developed to detect the onset of changes in systolic blood pressure; in [8], a preprocessing algorithm is proposed which provides a basis for an online trend extraction methodology [9]. An overview of alarm algorithms in critical care monitoring is given in [10].

**Figure 1:**
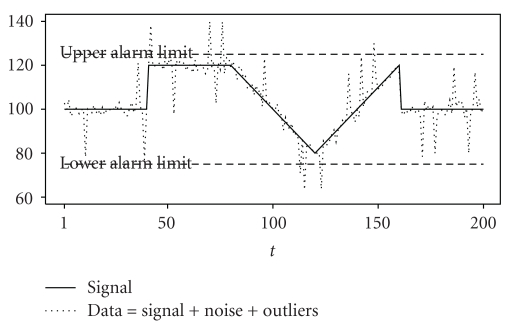
Generated time series data (dotted) consisting of the true signal (solid) overlaid with noise and outliers. Although the signal is within the alarm bounds (dashed), outliers would cause several unnecessary threshold alarms.

One approach to decreasing the false alarm rate is statistical signal extraction or filtering. Assume that the data consist of a true but unknown relevant signal overlaid with irrelevant noise and outliers. The signal can then be extracted *online*, meaning that the signal is extracted sequentially with each new incoming measurement. Then, the alarm limits can be compared to the online extracted signal instead of to the raw measured data, leading to fewer threshold alarms.  Figure 1 illustrates this approach. 

Time series from clinical online monitoring are not stationary but show level shifts, enduring and changing trends, a high level of noise, and are corrupted by (patches of) outliers. A suitable approach for online signal extraction, given such difficult data, is robust linear regression in a moving time window [11–13]. Then, the level of the regression line at the central or, alternatively, rightmost window position is used as the signal estimation. Using the level at the rightmost window position has the advantage that in an online application, the signal is estimated at the current time point, that is, without a time delay. We will, therefore, consider this type of moving window regression.

Since outliers appear frequently, robust regression methods should be applied. The robust Repeated Median (RM) regression [14] has proven to be a suitable candidate for the special situation of clinical online monitoring [11, 13]. It outperforms other robust regression methods with respect to robustness, computing time, and efficiency, in the sense of low bias and variability. However, as for any moving window technique, the RM signal estimation is strongly affected by the window size: large windows induce “smooth” signal estimations with little variability whereas small windows lead to signal estimations that are closer to the data. The *adaptive online RM* (aoRM) [15] chooses the window width for the RM automatically; as long as the data are “stable”, the window width gradually grows but when a structural change, for example, a level shift, occurs, the window width is set to a predetermined minimum value. The aoRM is enhanced to a filtering procedure for multivariate time series, namely, the *adaptive online Trimmed Repeated Median-Least Squares* (aoTRM-LS) filter [16]. This procedure factors in local cross-correlations (e.g., systolic and diastolic arterial pressure are highly correlated) in order to improve the filtering outcome.

A good filtering procedure suppresses “many” irrelevant (i.e., false) alarms while suppressing only “a few” relevant (i.e., true) alarms. In this paper, we will investigate aoRM and aoTRM-LS with respect to these criteria. The investigation is done using recorded monitoring data from an intensive care unit; all given alarms have been annotated retrospectively by an experienced physician as true or false. The aoRM and aoTRM-LS filters are applied offline to the data in order to determine the number of suppressed false alarms and the number of correctly reproduced true alarms. The corresponding performance criteria are *sensitivity* and *false alarm reduction rate*.

In the next section, we explain the aoRM and aoTRM-LS algorithms.  Section 3 describes the study setting, focussing on data collection and annotation. In Section 4, we illustrate the considered performance criteria and the way we determine them.  Section 5 presents the results of our analysis. A summary and an outlook are given in Section 6.

## 2. Signal Extraction Algorithms

The monitoring of *k* vital signs (e.g., heart rate, oxygen saturation, and systolic, diastolic, and mean arterial pressure) leads to a *k*-variate time series y(*t*) = (*y*_1_(*t*),…,*y*_*k*_(*t*))^T^, where *t* ∈ *ℕ* is the time index, indicating each second, for instance. The online signal extraction approach is based on the assumption that the observed data **y**(*t*) can be decomposed into a true but unknown signal which is overlaid with noise and outliers

(1) $$\text{y}\left(t\right)=\;\mu \left(t\right)+\varepsilon \left(t\right)+\eta \left(t\right)\in {\mathbb{R}}^{k}.$$

Here, *μ*(*t*) = (*μ*_1_(*t*),…,*μ*_*k*_(*t*))^T^ denotes the *k*-dimensional signal at time *t*. The noise term is *ε*(*t*), where *ɛ*_1_(*t*),…, *ɛ*_*k*_(*t*) are errors with zero median and time-dependent variances *σ*_1_(*t*),…, *σ*_*k*_(*t*). The errors may be correlated, that is, possibly Cov(*ɛ*_*i*_(*t*), *ɛ*_*j*_(*t*)) ≠ 0 for any *i* ≠ *j*. An outlier generating mechanism that produces impulsive spiky noise is denoted by *η*(*t*).

Both the aoRM and aoTRM-LS can be used to extract the signal vector *μ*(*t*) online: with each incoming new observation at time *t*, a new signal estimation output $\hat{\mu }(t)$ is produced. The aoRM is developed for univariate time series, meaning that it must be applied separately to each univariate component of a *k*-variate time series. On the contrary, the aoTRM-LS filter is developed for multivariate time series. It takes into account local dependences between variables and ensures robustness against outliers regarding the local covariance structure. Since aoTRM-LS is based on aoRM, we introduce the univariate aoRM first.

### 2.1. The Adaptive Online Repeated Median Filter (aoRM)

In [15], it is assumed that the underlying signal *μ*(*t*) of a univariate time series is approximately linear in a small moving window {*t* − *n* + 1,…, *t*} of length *n*

(2) μ(t−n+s)≈μ(t)+β(t)·(s−n),

where *s* = 1,…, *n* and *n* ≤ *t*. Here, *μ*(*t*) is the level of the straight line at the rightmost time point *t*, and *β*(*t*) is the associated slope in the time window. The rightmost window time point *t* corresponds to the current time point in the online case. Then, the RM estimates of *μ*(*t*) and *β*(*t*) in (2) are

(3) $$\begin{array}{cc} & {\hat{\beta }}^{\text{RM}}\left(t\right)\\ & =\underset{s\in \{1,\ldots ,n\}}{\text{med}}\left\{\underset{v\neq s,v\in \{1,\ldots ,n\}}{\text{med}}\left\{\frac{y\left(t-n+s\right)-y\left(t-n+v\right)}{s-v}\right\}\right\},\\ & {\hat{\mu }}^{\text{RM}}\left(t\right)=\underset{s\in \{1,\ldots ,n\}}{\text{med}}\left\{y\left(t-n+s\right)+{\hat{\beta }}^{\text{RM}}\left(t\right)\cdot \left(s-n\right)\right\}. \end{array}$$

The aoRM estimates the signal by the RM, after the window width has been adapted to the current data situation. The window width adaption is done automatically when a new observation comes in, that is, at each time point *t*. The adapted window width at time *t* is, therefore, denoted by *n*(*t*). After *n*(*t*) is determined, the signal *μ*(*t*) is estimated by RM regression in the time window {*t* − *n*(*t*) + 1,…, *t*}.

**Figure 2:**
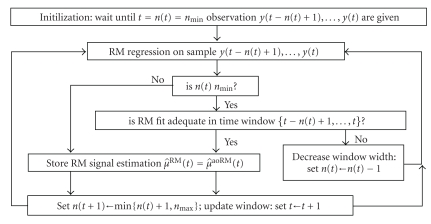
The aoRM algorithm.

In [15], it is demanded that *n*(*t*) ∈ {*n*_min_,…, *n*_max _}. The minimum width *n*_min_ guarantees robustness against a certain number of outliers while the maximum width *n*_max_ limits the computing time. Both *n*_min_ and *n*_max_ must be set beforehand by the user. A flow chart for the complete aoRM algorithm is shown in Figure 2. The main step of the aoRM algorithm is the decision of whether or not the RM fit in the time window {*t* − *n*(*t*) + 1,…, *t*}, that is, on the window sample *y*(*t* − *n*(*t*) + 1),…, *y*(*t*), is adequate. This decision is made by means of a test procedure which is based on the fact that an RM regression results in an equal number of positive and negative residuals. If the positive and negative residuals are not balanced for the *m* rightmost residuals within the time window of width *n*(*t*), the RM fit is regarded as inadequate. The input parameter *m* should be chosen such that *m* ≤ *n*(*t*)/2. For more details regarding the test procedure and the choice of *m*, see [15].

**Figure 3:**
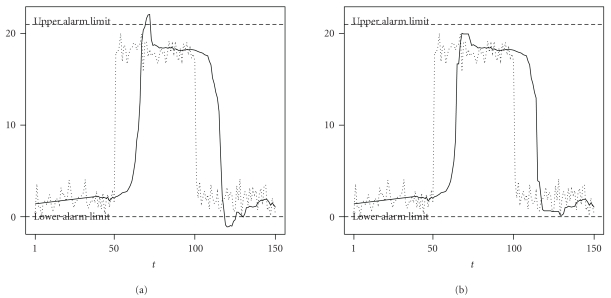
(a) aoRM signal estimations (solid) overshoot after sudden changes in the data (dotted). (b) effect of the restrict-to-range rule (5).

The aoRM filter estimates the signal at the rightmost or current time point, meaning that the signal is extracted without relevant time delay. (Its computing time depends on the chosen input parameters. The R package robfilter [17] provides a function of the aoRM; see Section 5.2. We applied this aoRM function using a 2.3 GHz computer with 2 GB RAM and obtained a mean computing time of 0.007 seconds for one iteration, resp., time point.) However, estimating the signal by the level at the right end of the regression line implies that the signal estimates possibly deviate distinctly from the data, especially when level shifts occur. Then, signal estimates “overshoot”; that is, they leave the range given by the window observations, see Figure 3(a). It shows a generated time series (dotted) with upwards and downwards level shifts at time points *t* = 50 and *t* = 100 and the corresponding aoRM signal estimation time series (solid). Around time *t* = 70 and *t* = 120, aoRM signal estimations overshoot. Those overshoots are crucial in our context since a sudden change in the data may cause the signal estimations to cross an alarm limit *although the measurements do not*, as can be seen in the figure. That is, an aoRM-based alarm system could theoretically cause more false alarms than a system based on raw measurements.

In order to prevent signal estimations from overshooting and causing false alarms, it is suggested in [15] to restrict the signal estimation ${\hat{\mu }}^{\text{aoRM}}\left(t\right)$ to a value within the range of the *m* most recent observations *y*(*t* − *m* + 1),…, *y*(*t*). (Note that this is the subsample which is used for the decision of whether or not the RM fit is adequate.) Defining the minimum and maximum of the *m* most recent observations by

(4) $$\begin{array}{cc} y_{m}^{\min\;}\left(t\right) & :=\min\;\;\left\{y\left(t-m+1\right),\ldots ,y\left(t\right)\right\},\\ y_{m}^{\max\;}\left(t\right) & :=\max\;\;\left\{y\left(t-m+1\right),\ldots ,y\left(t\right)\right\}, \end{array}$$

the *restrict-to-range rule* is

(5) $$\text{set}\;{\hat{\mu }}^{\text{aoRM}}\left(t\right)⟵\{\begin{array}{cc} y_{m}^{\min\;}\left(t\right), & \text{if}\;{\hat{\mu }}^{\text{aoRM}}\left(t\right)<y_{m}^{\min\;}\left(t\right),\\ y_{m}^{\max\;}\left(t\right), & \text{if}\;{\hat{\mu }}^{\text{aoRM}}\left(t\right)>y_{m}^{\max\;}\left(t\right). \end{array}$$

The effect of this rule can be seen in Figure 3(b). The signal estimations around time *t* = 70 and *t* = 120 are “pulled back” to the measurements. They do not violate the alarm limits, and, therefore, unjustified alarms are prevented. 

Figure 3 also shows that aoRM signal estimations trace changes in the data time series with a certain time delay. This inert reaction to sudden data changes is due to the RM's robustness against outliers. It has a finite sample replacement breakdown point of 50% [18]. That is, in order for a patch of level-shifted observations to affect the RM, the patch must consist of more than half of the window observations. Since a sudden change in the data implies that the window width is set down to *n*_min_, the choice of *n*_min_ is crucial: it defines the distinction between outlier patches and level shifts as well as the *tracing delay* for structural changes in the data, which is approximately *n*_min _/2 time points. In Figure 3, *n*_min_ = 40, so the tracing delay is approximately 20 time points.

The aoRM can be used for filtering multivariate time series by applying it separately to each univariate component time series. However, it does not account for dependences between the variables. The aoTRM-LS [16] is developed as a multivariate enhancement of the aoRM, which uses information given by the local covariance structure.

### 2.2. The Adaptive Online Trimmed Repeated Median-Least Squares Filter (aoTRM-LS)

The aoTRM-LS arises out of a combination of the aoRM and the multivariate TRM-LS [19]. Similarly to (2), it is assumed that each component of the underlying *k*-variate signal *μ*(*t*) is approximately linear in a short moving time window {*t* − *n* + 1,…, *t*}

(6) $$\mu \left(t-n+s\right)\approx \mu \left(t\right)+\beta \left(t\right)\cdot \left(s-n\right)\in {\mathbb{R}}^{k},$$

where *s* = 1,…, *n* and *n* ≤ *t*. Here, *μ*(*t*) is the level vector at the rightmost or current time point *t*, and *β*(*t*) the vector of the *k* slopes; see (2).

The aoTRM-LS filter can be used to estimate *μ*(*t*) and *β*(*t*) in (6). At each time *t*, the aoTRM-LS algorithm searches for an *overall window width n*_ov_(*t*) ∈ {*n*_min_,…, *n*_max _} which is adequate for all of the *k* variables. That is, it searches for the greatest *n*_ov_(*t*) such that the linear approximation (6) with *n* = *n*_ov_(*t*) is adequate. This search is done using the aoRM window width adaption principle. Thereafter, within the time window specified by *n*_ov_(*t*), the signal vector *μ*(*t*) is estimated by means of multivariate TRM-LS regression which is explained later on. The aoTRM-LS algorithm is as follows:

(0) initialization: wait until *t* = *n* = *n*_min_ observations *y*_*i*_(*t* − *n* + 1),…, *y*_*i*_(*t*), *i* = 1,…, *k*, are present,
(1) apply the aoRM window width adaption procedure to each of the *k* window samples *y*_*i*_(*t* − *n* + 1),…, *y*_*i*_(*t*) in order to obtain *k* appropriate *individual window widths n*_*i*_(*t*) ≤ *n*, and set the *overall window width n*_ov_(*t*) ← min _*i*_ {*n*_*i*_(*t*)};
(2) perform TRM-LS regression on the multivariate sample y(*t* − *n*_ov_(*t*) + 1),…, y(*t*), where y(·) = (*y*_1_(·),…,*y*_*k*_(·))^T^, and store the TRM-LS signal estimation ${\hat{\mu }}^{\text{TRM-LS}}\left(t\right)=:{\hat{\mu }}^{\text{aoTRM-LS}}\left(t\right)$,
(3) set *n* ← min  {*n*_ov_(*t*) + 1, *n*_max _}, and update the window: set *t* ← *t* + 1 and go to step (1).

After the overall window width *n*_ov_(*t*) is determined at step (1), the signal is estimated by means of TRM-LS regression at step (2). An outline of the TRM-LS regression algorithm is as follows (for simplicity, we set n* = *n*_ov_(*t*)).

First, RM regression is performed separately on each of the *k* window samples *y*_*i*_(*t* − n* + 1),…, *y*_*i*_(*t*). Then the RM residuals are regarded as a multivariate (*k* × n*)-sample *r*(*t* − n* + 1),…, r(*t*), where r(·) = (*r*_1_(·),…,*r*_*k*_(·))^T^. The local error covariance matrix Σ(*t*) ~ (*k* × *k*) is then estimated on this residual sample using a robust estimator proposed in [20]. The estimate $\hat{\Sigma }\left(t\right)$ is utilised to detect residual vectors that are outliers regarding the local covariance structure, that is, residual vectors r(*t* − n* + *s*), *s* = 1,…, n*, with

(7) $$\text{r}{\left(t-n^{*}+s\right)}^{\text{T}}\hat{\Sigma }{\left(t\right)}^{-1}\text{r}\left(t-n^{*}+s\right)>d,$$

where *d* > 0 is an adequate upper bound. (For more details see [16, 19] or [20].) Then, observation vectors y(*t* − n* + *s*), which correspond to outlying residual vectors r(*t* − n* + *s*), are removed from the window sample. Finally, a multivariate LS regression is performed on the outlier-free window sample, and the levels at the right end of the *k* LS regression lines build the signal estimation vector.

The aoTRM-LS yields robust but also efficient signal estimations since the signal vector is finally estimated by means of LS regression. Just like aoRM, aoTRM-LS estimates the signal vector *μ*(*t*) at the right end point of the moving time window. Hence, the restrict-to-range rule (5) is also recommended for aoTRM-LS. Furthermore, for data that exhibit a known correlation structure, it is suggested to apply the aoTRM-LS filter not to the whole *k*-variate time series but separately to blocks that consist of highly positively correlated variables [16]. For instance, systolic, mean, and diastolic blood pressure are highly positively correlated and, therefore, can be combined into a *correlation block*. A block wise application of aoTRM-LS improves the window width adaption, leading to smoother signal extraction time series.

We apply both aoRM and aoTRM-LS retrospectively to recorded online-monitoring data from intensive care in order to evaluate their ability to suppress false and to reproduce true alarms. In the following, we describe the study setting, including data collection and annotation.

## 3. Study Setting

In accordance with the declaration of Helsinki, the study was approved by the Ethics Committee of the University of Regensburg. The data were collected at an intensive care unit at the University Hospital Regensburg [21]. Only adult patients with continuous monitoring of at least invasive arterial blood pressure, heart rate, and oxygen saturation were included into the study. The deployed monitoring system was an Infinity Monitor by Dräger Medical, Lübeck, Germany. Data acquisition from the Infinity Monitoring System took place using the special software eData by Dräger Medical, Lübeck, Germany. The data were recorded at a 250 Hz sampling rate, labeled with a time index, and stored. The reference data that we consider were extracted from these data at a sampling rate of one per second and stored in text files. This reference database includes

- numerical measurements of the vital signs,
- all monitoring system alarms with corresponding time and alarm message (e.g., “heart rate lower limit violation”),
- the alarm limits that were set by the medical staff,
- information when alarms were deactivated (“alarms off” periods).

For more details about the database, see Section 5.1.

All alarm situations were retrospectively annotated by an experienced physician by means of graphical representations of the collected data from the monitoring system in combination with video recordings showing the patient and the screens of the monitoring system. Since the data contain the alarm time points, the physician could wind the video tape and watch the patient and the screens of the monitoring system at these alarm time points. Then, by means of a specially developed JavaScript program [22], each alarm situation was assessed in terms of whether it was clinically relevant and whether it was technically true.

In this study, an alarm is regarded as *technically true* if it is based on a correct measurement or if the monitoring system correctly recognizes a technical problem and gives a technical alarm. All other alarms are *technically false*. Furthermore, a situation is defined as *alarm relevant* or *true*, if it implicates a diagnostic or therapeutic decision or the correction of a technical problem. A technically true alarm is annotated as *advisory* if it does not require immediate action; that is, it is not alarm relevant but judged to be helpful. All other alarms are annotated as *not alarm relevant* or *false*, respectively. A technically false alarm is always a false alarm.

## 4. Performance Criteria

Our aim is to evaluate the “new” aoRM and aoTRM-LS alarm systems and compare their performances to that of the “old” alarm system based on raw measurements. In this context, an alarm system is a diagnostic tool: an alarm corresponds to the diagnosis “alarm relevant situation”. Common performance criteria for diagnostic methods are sensitivity (SE) and specificity (SP). In our context, SE is the conditional probability of an alerting monitoring system given that the situation is actually alarm relevant, and SP is the conditional probability of a non-alerting monitoring system, given that the situation is actually not alarm relevant

(8) $$\begin{array}{c} \text{SE}=P\left(\text{alarm}\;\text{given}\;|\;\text{alarm}\;\text{relevant}\;\text{situation}\right),\\ \text{SP}=P\left(\text{no}\;\text{alarm}\;\text{given}\;|\;\text{not}\;\text{alarm}\;\text{relevant}\;\text{situation}\right). \end{array}$$

While SE assesses the performance with respect to the detection of alarm relevant situations, SP quantifies the liability of the alarm system to produce false alarms. Both SE and SP of a diagnostic method should be large; at best, both equal to 1.

The common approach to estimate an alarm system's SE is to determine the ratio of detected alarm relevant situations and all alarm relevant situations. In our study, alarms given by the old system (*positive alarms*) were annotated as either *true positive* or *false positive*. Nongiven alarms, that is, *negative alarms*, including *false* negative alarms, do not occur. Conversely, this means that the old system detects all alarm relevant situations and, therefore, has 100% SE. However, this is not de facto but follows from the study design.

The SE of a new alarm system based on signal filtering can be estimated by the ratio of the number of alarm relevant situations that are detected, that is, reproduced correctly, and the number of all alarm relevant situations. A situation is regarded as detected by a new system if the signal estimations violate the alarm limits close to the time when the alarm was given by the old system. A detailed description is given later on.

Similar to SE, SP is usually estimated by the ratio of the number of *true negative* alarms and the number of *not* alarm relevant situations. However, due to the study design, there is no information about these numbers. Therefore, in [23], an alternative approach for estimating the SP of an online-monitoring alarm system is used: defining FP_max_ as the highest expected number of false positive alarms and FP as the actual number of false positive alarms, the difference FP_max_ − FP is an estimate for the number of true negative alarms. This number is then divided by the worst case number FP_max_ to get an alternative estimation of SP:

(9) $$\hat{\text{SP}}=\{\begin{array}{cc} \frac{{\text{FP}}_{\max\;}-\text{FP}}{{\text{FP}}_{\max\;}}, & \text{FP}\leq {\text{FP}}_{\max\;},\\ 0, & \text{FP}>{\text{FP}}_{\max\;}. \end{array}\;$$

**Figure 4:**
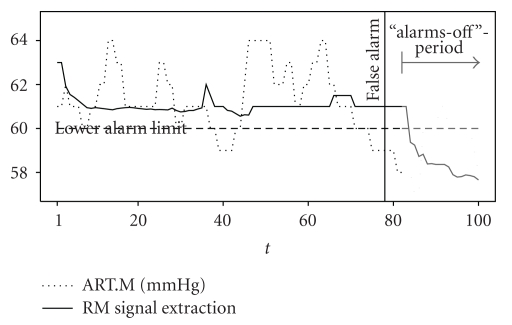
Measurements of mean arterial blood pressure (ART.M, dotted) and RM signal estimations (solid).

For SP estimation as proposed in [23], the analyst has to decide whether or not to count in alarms of the new system that occur during “alarms off” periods. In these periods, alarms were deactivated by the clinical staff—even if the measurements violated the alarm thresholds, no alarm was given. Since alarms are often deactivated as a reaction to threshold alarms, a new system would be favoured if it cannot produce alarms in these periods; see Figure 4. It shows a part of a time series of mean arterial blood pressure measurements (dotted) and the corresponding signal estimations by the simple RM with window width *n* = 60 (solid). Due to the RM's robustness against outliers, its signal estimations react to changes in the data with a tracing delay of approximately *n*/2 = 30 time points, as explained in Section 2. Since the staff deactivated all alarms at *t* = 82, only four seconds after the false alarm at *t* = 78 occurred, the signal extraction time series violates the lower alarm limit within the “alarms off” period. If “alarms off” periods were not considered in the analysis, the false alarm at *t* = 78 would be regarded as suppressed by the new system although its signal estimations violate the lower alarm limit. Thus, in order to not favour the new system, one must include alarms of the new system that would be given during “alarms off” periods. However, during those periods the old system cannot produce threshold alarms, but the new system *can*. Moreover, each alarm of the new system is regarded as false if it does not belong to a situation annotated as relevant. That is, within “alarms off” periods, each limit violation of a new system would be regarded as false alarm. In a nutshell, due to the given study design and data basis, for SP estimation the analyst must decide in favour of the old or new system.

Due to these considerations, we refrain from determining SP. Instead, we consider the ratio of false alarms, which are *suppressed* by the new system, to all false alarms, denominated as *false alarm reduction rate* (FARR) of the new system:

(10) $$\text{FARR}=\frac{\#\;\text{suppressed}\;\text{false}\;\text{alarms}}{\#\;\text{false}\;\text{alarms}}.$$

Similarly to the detection of true alarms, a false alarm is regarded as *suppressed* by the new system if its signal estimations *do not* violate the alarm limits close to the time when the false alarm occurs.

Obviously, the old alarm system has a FARR of 0%; that is, the new system cannot be worse with respect to this criterion. Moreover, there might be alarms of the new system that are not regarded. Hence, alarms of the new system should rather be considered independently from false alarms of the old system.

However, the restrict-to-range rule (5) guarantees that the estimated signal only violates the upper (e.g.) alarm limit, if at least one of the *m* most recent measurements also violates the upper limit. Thus, an aoRM/aoTRM-LS alarm system cannot generate more alarms than the old system. However, the old system already includes a simple algorithm to suppress false alarms. It gives an alarm only if observations lie outside the alarm limits for a certain time span, the *alarm validation time*; see Table 1. For instance, an alarm caused by a too high systolic blood pressure is not given until four consecutive measurements are above the upper alarm limit whereas a single heart rate measurement below the lower alarm limit causes an alarm immediately. 

Because of alarm validation times, aoRM/aoTRM-LS signal estimations *can* violate an alarm limit (and hence produce an alarm) although the old system has not given an alarm beforehand. However, such cases are very rare and occur only if measurements fluctuate around an alarm limit. In those cases, an alarm is rather helpful: either the alarm limits are set too narrow, or the patient's condition deteriorates.

Due to these considerations and taking into account the fact that SP does not allow for a fair comparison, we conclude that FARR is a more sensible performance criterion in our situation.

In the following, we explain how we estimate SE and determine the FARR of a new alarm system based on signal extractions. For simplicity, SE now denotes the estimated sensitivity.

As mentioned above, SE of a new alarm system can be determined by the ratio of detected true alarms and all true alarms. A true alarm can be regarded as detected if the signal estimations violate the alarm limits close to the time when the true alarm occurred. Hence, we consider all *true alarm time points t*_*i*_^true^, *i* = 1,…, *M*, which correspond to true threshold alarms of the old system. If signal estimations violate the alarm limits in a certain time range in proximity to *t*_*i*_^true^, the referring true alarm is regarded as detected by the new alarm system.

In contrast to SE, FARR is determined using all alarm time points which are annotated as *false*, that is, *t*_*j*_^false^, *j* = 1,…, *N*. If aoRM/aoTRM-LS signal estimations *do not* violate the alarm limits in a certain time range in proximity to *t*_*j*_^false^, the corresponding false alarm is regarded as *suppressed* by the new alarm system. Then, the FARR of the new system is the ratio of suppressed false alarms to all false alarms.

In order to specify the range around true alarm time points *t*_*i*_^true^, we build *true alarm intervals *{*t*_*i*_^true^ − *D*,…, *t*_*i*_^true^ + *D*}. The *detection tolerance time D* was chosen by the physicians involved in the study. For ART.M, ART.S, and SpO_2_, this time is *D* = 60, and for HR it is *D* = 30 seconds. However, we decided to build true alarm intervals which merely include *D* time points *after* an alarm time point, that is, {*t*_*i*_^true^,…, *t*_*i*_^true^ + *D*}, since aoRM and aoTRM-LS were developed for suppressing false alarms. They do not forecast alarm situations but react to the data which is reflected in the tracing delay. Thus, including time points left from *t*_*i*_^true^ into the alarm interval makes little sense.

Similar to true alarms, the range after a false alarm *t*_*j*_^false^ is specified by a *false alarm interval *{*t*_*j*_^false^,…, *t*_*j*_^false^ + *S*}. The *suppression tolerance time S* must be chosen such that it is greater than the tracing delay of the signal estimations. This prevents false alarms from being assessed as suppressed by the new system just because its signal estimations violate the alarm limits too late. The largest minimum window width is *n*_min_ = 90 in our study, which corresponds to a tracing delay of approximately 45 seconds. Hence, we think that *S* = 60 is an ample suppression tolerance time.

The determination of SE and FARR based on true and false alarm intervals does not work without further complications: we have to bear in mind that some false alarms appear close to true alarms so that the corresponding alarm intervals overlap. Thus, there might be inconsistencies since a new alarm could be regarded as detected true alarm *and* as not suppressed false alarm. Since the detection of true alarms is more crucial than the suppression of false alarms, we simply exclude those false alarms from the analysis whose false alarm intervals overlap with true alarm intervals. Hence, approximately 10% of false alarms are excluded from the analysis.

## 5. Evaluation of the aoRM and aoTRM-LS Alarm Systems

In this section, we first analyse the database and introduce a strategy to handle advisory alarms; that is, alarms that were not alarm relevant but helpful. Then, the results of our analysis regarding SE and FARR of the new aoRM and aoTRM-LS alarm systems are presented.

### 5.1. The Database and Reassessment of Advisory Alarms

Our reference database contains recorded online-monitoring data from 85 different cases between January 2006 and May 2008. A case stands for one disease episode of a patient, meaning that some patients correspond to several cases.

The overall monitoring time is 1245:52:28 hours, and the mean monitoring time for each case is 14:39:26 hours (minimum 0:55:01 hours, maximum 31:35:25 hours). The monitoring system generated a total number of 9290 alarms, of which 4825 (52%) were simple threshold alarms. A total number of 9290 alarms means a frequency of 7-8 alarms per hour. However, considering the 85 cases, the-alarm-per hour frequency was quite dissimilar. Furthermore, the alarm frequency is higher in the morning and the afternoon, which is probably related to nursing actions during the day.

Several vital parameters were monitored like the heart rate, pulse, blood oxygen saturation and temperature, respiratory rate, and systolic, mean, and diastolic artery blood pressure. However, only mean artery blood pressure, heart rate, and blood oxygen saturation were monitored in each case.

Since 90% of all threshold alarms are caused by systolic and mean artery blood pressure (ART.S and ART.M), heart rate (HR), and oxygen saturation (SpO_2_), our analysis concentrates on these four vital signs. The numbers of true, false, and advisory threshold alarms regarding ART.S, ART.M, HR, and SpO_2_ are listed in Table 2. 

Only 10% of all alarms were true whereas 43% were false and 47% advisory alarms. This low rate of relevant alarms matches other studies; see [10]. (All alarms means all alarms given by the four considered vital signs in the following.) The most alarms were produced by ART.S (42%). This vital sign exhibits a lower rate of false alarms but more advisory alarms than average. SpO_2_ produced 26% of all alarms. Its false alarm rate is highest, and it has the lowest proportion of advisory alarms. For HR, which produces 21% of all alarms, the opposite is true: most HR alarms were advisory. ART.M produced the least alarms (11%), of which 11% were true, 49% advisory, and 40% false alarms.

Advisory alarms are problematic, since our analysis is based on true/false decisions. One possibility is to simply exclude advisory alarms from the analysis. However, this approach is not really satisfying since nearly half the alarms were advisory, for heart rate even more than three-quarters. Too many situations would be excluded, so the informative value of the analysis would be lowered substantially. Moreover, advisory alarms also involve an alarm sound and, therefore, need to be considered. We, therefore, decide to assess each individual advisory alarm as either true or false. The assessment is done regarding the *alarm length*, which is the time an alarm is active, that is, the time span for which the measurements lie outside the alarm limits. The idea is that short alarm limit violations do not exhibit clinical relevance and are interfering rather than helpful [24, 25]. According to the physicians involved in the study, advisory alarms of ART.S and ART.M that are shorter than 10 seconds can be regarded as irrelevant. An advisory HR or SpO_2_ alarm is regarded as irrelevant if it is shorter than 5 or 15 seconds, respectively. Using this strategy, the set of 2032 advisory alarms is split into 1201 true and 831 false alarms. That is, there are 1650 (38%) true and 2709 (62%) false alarms now. However, due to overlapping alarm intervals (see Section 4), we exclude 418 false alarms from the analysis so that we obtain 1650 (42%) true and 2291 (58%) false alarms (Table 3). The highest false alarm rate is still produced by SpO_2_ with 79% irrelevant alarms. ART.S exhibits a 55% and ART.M a 51% false alarm rate. HR has the lowest false alarm rate (39%). 

Due to the reassessment of advisory alarms, we are able to perform the analysis regarding SE and FARR as described in Section 4. In the following, we explain how aoRM and aoTRM-LS are applied to the data. Afterwards, we present the results.

### 5.2. Application of aoRM and aoTRM-LS

For the offline application of aoRM and aoTRM-LS to the recorded monitoring data, we use the open source software R, version 2.10.1. The R package robfilter [17] contains functions of aoRM and aoTRM-LS, the function names are adore.filter (equates to aoRM) and madore.filter (equates to aoTRM-LS).

Using R, one can handle a broad range of data storage formats, also including the text file format of the reference data. Each of the 85 cases refers to one data set, that is, one text file. Each data set text file is loaded in R in form of a data matrix with *T*_*i*_ rows, *i* = 1,…, 85, where each column contains the measurements of one vital parameter. The number of rows *T*_*i*_ equals the monitoring time in seconds of case *i*.

The adore.filter function is designed for univariate time series which correspond to vectors in R. Hence, the adore.filter function is applied column by column. In contrast, the madore.filter function is designed for multivariate time series and can, therefore, be applied to a whole data matrix. The signal estimation outputs of the adore.filter and madore.filter functions are stored in text files, so that they can be analysed regarding SE and FARR as explained in Section 4. This analysis is also done by means of R 2.10.1.

For an application of the filters in clinical practice, the treatment of missing values is an issue we are concerned with: due to technical problems, online-monitoring data can contain missing values at single points as well as long stretches of missing values. The functions adore.filter and madore.filter have similar strategies to deal with missing values. Their algorithms give an output only if enough observations for a reasonable signal estimation are present. Otherwise, the signal estimation output is a missing value. For more details, see the R help or [15, 16].

Since the monitored vital signs hold a block dependence structure (cf.  Section 2.2), we apply aoTRM-LS block wise to one block consisting of ART.S, ART.M, and diastolic artery blood pressure, and to one block consisting of HR and pulse. SpO_2_ has an exceptional position since it is a “block” on its own. In this case, the madore.filter algorithm applies the univariate adore.filter meaning that the madore.filter and adore.filter signal estimations are equal for SpO_2_. Our analysis still concentrates solely on ART.M, ART.S, HR, and SpO_2_ although aoTRM-LS is also applied to diastolic artery blood pressure and pulse rate measurements—these measurements provide additional information for the aoTRM-LS filter.

As explained in Section 2, the minimum window width *n*_min_ is crucial, since it determines (a) the tracing delay of aoRM/aoTRM-LS signal estimations when reacting to sudden data changes and (b) the number of outliers the filtering procedures can resist. Therefore, we apply aoRM and aoTRM-LS to the whole data set using *n*_min_ = 10,20,…, 90. The width *m* of the time window, which is used to assess the RM regression fit and as comparison sample for the restrict-to-range rule, is always *m* = *n*_min _/2. The maximum window width is always *n*_max_ = 300.

### 5.3. Results

**Figure 5:**
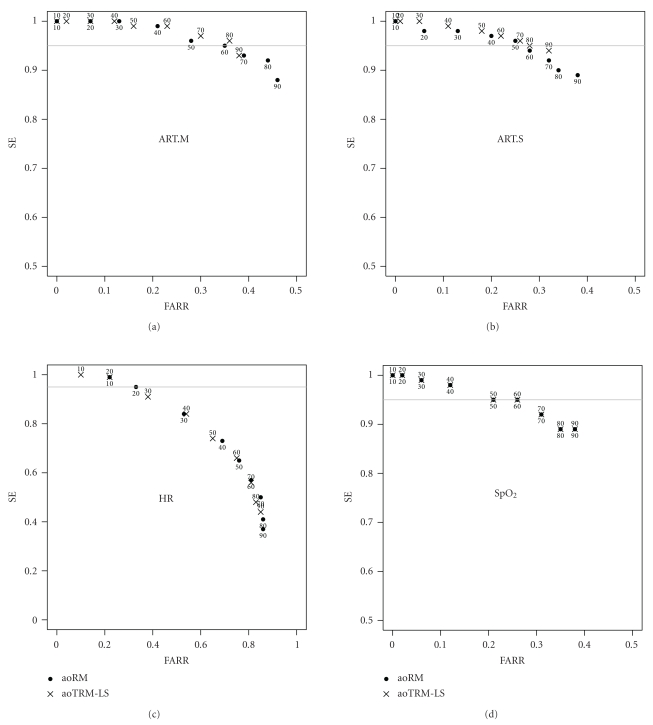
SE and FARR of aoRM and aoTRM-LS for each of the four vital signs. The number below (above) a dot (cross) indicates the used *n*_min_.

We determine SE and FARR of the aoRM and aoTRM-LS alarm systems separately for each of the four vital signs ART.M, ART.S, HR, and SpO_2_. In Figure 5, we plot FARR against SE for each vital sign and each minimum window width *n*_min_ = 10,20,…, 90. SE and FARR of aoRM are indicated by dots, SE and FARR of aoTRM-LS by crosses. The number below (above) a dot (cross) indicates the used minimum window width *n*_min_. Note that for HR the plot range is [0,1]×[0,1] whereas for the other vital signs it is [0,0.5]×[0.5,1]. As can be seen in Figure 5, the greater the *n*_min_, the greater the FARR and the smaller the SE. According to the physicians involved in the study, we demand at least 95% SE, marked by the grey lines in Figure 5. This helps with finding the *n*_min_ which induces the largest FARR under the restriction of at least 95% SE.

For ART.M, aoTRM-LS with *n*_min_ = 80 offers SE ≈ 96% and FARR≈36%. The aoRM filter with *n*_min_ = 60 yields comparable results with SE≈95% and FARR ≈35%. Regarding ART.S and demanding at least 95% SE, we obtain somewhat worse results with FARR≈25% for aoRM (*n*_min_ = 50) and FARR≈28% for aoTRM-LS (*n*_min_ = 80). In order to obtain at least 95% SE for HR, a smaller minimum window width must be chosen. Using aoRM with *n*_min_ = 20, approximately one-third of all HR false alarms can be suppressed with SE≈95%. Using the same minimum window width for aoTRM-LS, we obtain SE≈99% but merely FARR ≈22%; using *n*_min_ = 30 leads to FARR≈38% but with SE≈91%. Since SpO_2_ builds a block on its own, the multivariate madore.filter algorithm applies the univariate adore.filter to this block. Thus, the aoRM and aoTRM-LS signal estimations are equal for SpO_2_. Using *n*_min_ = 60 we obtain FARR≈26% and SE≈95%.

## 6. Summary and Outlook

The aoRM and aoTRM-LS signal filters can be used to extract signals online from nonstationary, noisy, and outlier contaminated online-monitoring time series from intensive care. By comparing signal estimates instead of raw measurements to upper and lower alarm limits, the number of threshold alarms can be reduced.

The evaluation of aoRM and aoTRM-LS for an application in clinical practice is done based on recorded data from an intensive care unit. In this data record, each alarm has been annotated retrospectively as either true or false. In order to evaluate the aoRM and aoTRM-LS alarm systems, we estimate their sensitivity (SE) but not their specificity. The reason is that for a specificity estimation one has to decide whether or not to regard limit violations of the new system within “alarms off” periods, meaning that either the new or old alarm system is favoured. Hence, we refrain from estimating specificity and determine the false alarm reduction rate (FARR) of the new system instead.

FARR and SE of the new system are determined by building false and true alarm intervals which consist of a certain number of time points after a false and true alarm, respectively. If the aoRM/aoTRM-LS signal estimations violate the alarm limits within a true alarm interval, the according true alarm is regarded as detected. Analogically, a false alarm of the old system is regarded as suppressed by the new system, if its signal estimations do not violate the alarm limits within the false alarm interval.

An analysis of the database shows that more than 90% of all threshold alarms were caused by ART.S, ART.M, HR, and SpO_2_. Hence, we concentrate our analysis on these four vital signs. Furthermore, almost half of all alarms were assessed as advisory; these alarms do not require immediate action but are helpful. Since our evaluation strategy is based on true/false decisions, we reassess advisory alarms as false or true.

The application of aoRM and aoTRM-LS is performed retrospectively using the R functions adore.filter and madore.filter from the R package robfilter. Since the choice of the minimum window width *n*_min_ has a great impact on the aoRM/aoTRM-LS signal extraction, we apply the filters using *n*_min_ = 10,20,…, 90. The function madore.filter (aoTRM-LS) has been applied block wise with one block consisting of arterial blood pressures and one of heart rate and pulse; oxygen saturation builds an own block, meaning that the multivariate madore.filter algorithm applies the univariate adore.filter to this block.

We found that both filters are able to suppress around a quarter to a third of all false alarms while providing at least 95% sensitivity. Using a larger minimum window width, a greater FARR can be obtained at the cost of a lower SE. For instance, merely demanding at least 90% SE, an aoRM or aoTRM-LS alarm system can reduce 31 to 38% of all threshold alarms.

The need for an improvement of the actual situation on intensive care units is obvious. Preprocessing the raw monitoring data by the aoRM or aoTRM-LS filter is a good possibility to achieve this aim. The proposed filters could be implemented into the monitoring systems. Then, the practical performance of a filtering based alarm system can be compared to that of the “old” system in a test phase. However, one has to keep in mind the lower sensitivity of filtering based alarm systems. Therefore, the physician must have access to both the filtered and the raw measurements at the bedside.

The potential of the aoRM and aoTRM-LS signal filters is not restricted to false alarm suppression. For instance, they can also be used to ease patient monitoring since smooth signal extractions are easier to interpret than noisy and outlier contaminated measurements. The aoRM and aoTRM-LS signal extraction can also be beneficial in other fields, for example, for high-frequency measurements from industry or finance.

Furthermore, the aoRM and aoTRM-LS can be enhanced or used as a basis for other filters. For instance, there might be improved principles for the window width adaption, possibly based on methods for the detection of structural breaks; for the aoTRM-LS, an automatic and time-dependent choice of the correlation blocks (see Section 2.2) would be an improvement, especially for multivariate time series with an unknown and possibly changing dependence structure.

## Figures and Tables

**Table 1:** Alarm validation time: measurements must violate the upper/lower alarm limit for a certain time in order that an alarm is given.

Vital sign	Upper alarm limit	Lower alarm limit
Heart rate	2 seconds	immediately
Blood pressure	4 seconds	4 seconds
Oxygen saturation	4 seconds	10 seconds

**Table 2:** Numbers of threshold alarms regarding ART.S, ART.M, HR, and SpO_2_. The percentages marked with * are the proportions of the individual true, advisory, and false alarm rates to all alarms of the respective individual vital parameter.

Annotation	ART.S	ART.M	HR	SpO_2_	Σ
True	189 (10%*)	54 (11%*)	71 (8%*)	135 (12%*)	449 (10%)
Advisory	981 (53%*)	237 (49%*)	704 (77%*)	110 (10%*)	2032 (47%)
False	680 (37%*)	195 (40%*)	134 (15%*)	869 (78%*)	1878 (43%)

Σ	1850 (42%)	486 (11%)	909 (21%)	1114 (26%)	4359 (100%)

**Table 3:** Numbers of threshold alarms regarding ART.S, ART.M, HR, and SpO_2_ after each advisory alarm has been assessed as true or false. The percentages marked with * are the proportions of the individual true and false alarm rates to all alarms of the respective individual vital parameter.

Annotation	ART.S	ART.M	HR	SpO_2_	Σ
true	735 (45%*)	215 (49%*)	481 (61%*)	219 (21%*)	1650 (42%)
false	908 (55%*)	228 (51%*)	309 (39%*)	846 (79%*)	2291 (58%)

Σ	1643 (42%)	443 (11%)	790 (20%)	1065 (27%)	3941 (100%)
